# Vulnerability assessment of food imports—Conceptual framework and empirical application to the case of Switzerland

**DOI:** 10.1016/j.heliyon.2024.e27058

**Published:** 2024-02-24

**Authors:** Christian Ritzel, Anke Möhring, Albert von Ow

**Affiliations:** a Agroscope, Research Group Economic Modelling and Policy Analysis, 8356, Ettenhausen, Switzerland; b FiBL, Research Group Evaluation and Impact Assessment, 5070, Frick, Switzerland

**Keywords:** Trade, Net importer, Principal component analysis, Exogenous shocks, Political crisis, Climate risks

## Abstract

The world's food supply is becoming increasingly vulnerable because of rising political and climatic crises. To identify food supply vulnerabilities at an early stage, this paper proposes a multifactorial and standardized import vulnerability index (IVI). The IVI encompasses (i) an exporter vulnerability index (EVI) consisting of four exporter-specific factors and (ii) a product vulnerability index (PVI) consisting of three product-specific factors. We applied a robust principal component analysis to derive weights to combine these individual factors into a standardized IVI. The IVI was applied to food import-dependent Switzerland from 2014 to 2019 as an empirical case study. The results showed that a large share of Swiss food imports mainly originated from neighboring countries, indicating a very low to moderate EVI. Regarding the PVI, only product groups that were imported from a small number of countries (e.g., oilseeds) or that showed a low self-sufficiency ratio (e.g., coffee) or high price volatility (e.g., meat) exhibited a high value. Moreover, the findings demonstrate that the IVI showed neither large fluctuations nor a decreasing or increasing trend. With regular updates, the proposed indicator can become a valuable monitoring tool for food supply security.

## 1 Introduction

Awareness of potential food supply vulnerabilities is critical for policymakers to ensure appropriate measures for food supply security. Due to the complex relationships in the agri-food sector, the COVID-19 pandemic led to long-lasting disruptions of global supply chains [1]. Recent findings indicate that the COVID-19 pandemic reduced food trade by 5–10% in 2020 [2]. Similarly, political instabilities can greatly affect the supply of food and production resources [3,4]. As one of the consequences of the Ukraine crisis, significantly reduced global export availability could drive other exporting countries to protect their domestic supplies by slowing or restricting exports [5]. A wide range of further permanent ongoing threats, such as climate change [6,7] and increasingly spreading plant or animal diseases [8,9], have increased the insecurity and volatility of global food supply chains. Although such hazards primarily negatively affect poorer countries' food security, a significant global shortage in the food supply can also threaten supplies in richer countries [10]. Especially for countries with a low food self-sufficiency ratio (SSR), interrupting supply chains has negative effects on food supply security. As one of the four dimensions of food security,^1^ food availability (i.e., food available for human consumption) is determined by a country's domestic food production and food imports [11]. To ensure food imports, reliable trade partners and stable supply chains are indispensable for these countries.

In the current paper, we use the concept of vulnerability. To the best of our knowledge, a holistic vulnerability assessment of food imports that combines theory and empirical evidence is missing in the literature. A country's vulnerability is defined by its degree of exposure to exogenous hazards or shocks, such as extreme climate events, unstable trade relations, and market price volatility [12]. To build an indicator system that assesses the vulnerability of food imports, exporter-specific and product-specific factors that potentially jeopardize food imports must be captured as comprehensively as possible by using readily available data. Accordingly, the main objective of this study is to develop a conceptual framework for a combined (multifactorial) and standardized import vulnerability index (IVI) that encompasses (i) an exporter vulnerability index (EVI) consisting of four exporter-specific factors and (ii) a product vulnerability index (PVI) consisting of three product-specific factors. Individual factors are identified and refined based on an extensive literature review of vulnerability assessments that refer to different application scopes, such as the whole economy, food security, the energy sector, and the defense-related industry. We apply a robust principal component analysis (PCA) to determine the weights for combining these individual factors into a standardized IVI. To demonstrate the applicability of the IVI, we use food import-dependent Switzerland as a case study (Gross food SSR in 2018: approximately 60%; [13]). The analysis is conducted at the product group level for the years 2014–2019. The products are classified according to the Harmonized System (HS) on the 2-digit level (the total 14 HS chapters considered for the empirical exercise can be found in Table 10 in the appendix).

We find that between 2014 and 2019, Switzerland's total food imports originated from a broad range of countries. However, the bulk of food imports originated from countries with low EVI values. This implies that Switzerland's main trade partners were low in vulnerability regarding governmental quality, climatic conditions, supply capacity, and technological capacity. Furthermore, to a large extent, Switzerland's food imports originated from neighboring countries.

For most of the considered product groups, the PVI value remained relatively stable from 2014 to 2019. Whereas the product groups “Milk, eggs, and honey” and “Vegetable preparations” showed the lowest PVI values, the product groups “Oilseeds” and “Mill products” showed the highest PVI values. In the case of “Oilseeds” and “Mill products”, PVI values were mainly determined by a high concentration of exporting countries. We observe a strong import dependency for products that cannot be produced in Switzerland (i.e., coffee and exotic fruits). For the product group “Meat,” which is imported in auctioned tariff rate quotas, we observe a high import price volatility. The overall IVI for Switzerland's food vulnerability exhibited neither large fluctuations nor trends from 2014 to 2019. In general, exporter-specific factors played a superordinate role compared with product-specific factors during this period. Although climate change increasingly negatively affects global agricultural production [14], surprisingly, the contribution of the climate-related factor to the IVI remained almost constant from 2014 to 2019.

Annual assessments using monitoring tools such as the IVI can reveal trends (i.e., increasing or decreasing vulnerabilities) that could inform policymakers and actors along the supply chain (i.e., importers, processors, and retailers) to intervene in a timely manner. Therefore, on the one hand, the IVI needs to meet scientific standards, such as transparency, traceability, and reproducibility; on the other hand, it must be simple regarding its communicability to policymakers. Studies assessing the vulnerability of food trade usually use single indicators, or various indicators that are nevertheless not combined to a standardized and composite vulnerability index. The proposed index might be of interest to other food import dependent countries beyond Switzerland (e.g., countries with a food SSR <85%), such as Japan, South Korea, Greece, Italy, Mexico, and Kuwait [15].

The remainder of the current article is organized as follows: In Section 2, we review the literature on vulnerability assessments. In Section 3, we provide the conceptual framework of the composite IVI, the underlying databases (subsection 3.1), and the method used for constructing the IVI (subsection 3.2). In Section 4, the empirical results are presented for the EVI (subsection 4.1), the PVI (subsection 4.2), and the overall IVI (subsection 4.3). In Section 5, we conclude the paper and provide policy implications.

## 2 Literature review on vulnerability assessments

Extant scientific studies on vulnerability assessments have focused on energy imports or energy supply (e.g., gas or oil), food security, the defense-related industry, or the whole economy of the country. These studies provide valuable insights and inspiration for the design of a multifactorial IVI in the food sector. Table 1 presents an overview of composite vulnerability assessment indices, their application scope (i.e., energy sector, whole economy, food sector, and defense-related industry), their individual factors (subindices), and their value ranges, as well as the methods employed for constructing these composite vulnerability indices.

**Table 1:** Literature review on vulnerability assessment indices.

Index and source	Application scope	Factors/subindicators	Value range	Applied method
Oil vulnerability index [16]	Energy sector	Ratio of value of net oil imports to gross domestic product Oil consumption per unit of GDP GDP per capita Oil share of total energy supply Ratio of domestic reserves to oil consumption Exposure to geopolitical oil market concentration risks as measured by net oil import dependence Ratio of world oil supply to oil demand (market liquidity)	Least vulnerable (average value = 0.389); less vulnerable (average value = 0.562); more vulnerable (average value = 0.810); most vulnerable (average value = 1.003)	Principal component analysis
Economic vulnerability index [17]	Economy	Import vulnerability (import concentration, product type, country of origin) Export vulnerability (export concentration, product type, country of destination) Foreign direct investment vulnerability (lack of foreign direct investment potential, country of origin)	0 = minimum vulnerability; 1 = maximum vulnerability	Subindicators are weighted by further variables and standardized
Bonilla index [18]	Food sector	Value of total food imports (quantity of food imports times world price in foreign currency) compared with Value of total exports (quantity of exports times export price in foreign currency)	Low or moderate Bonilla index = 1–10%; high Bonilla index = 11–100%; Bonilla index >100% possible	No weighting and standardization
Trade vulnerability index [19]	Food sector	Import dependence: Amount of China's grain imports from a given exporter divided by the sum of China's grain imports from all exporters Export dependence: Amount of China's grain exports to a given importer divided by the sum of China's grain exports to all importers	The values of import and export dependence range between 0 and 100% The vulnerability index has the following levels: low vulnerability (-∞, 0.7); medium vulnerability (0.7, 1.6); high vulnerability (1.6, +∞)	No weighting and standardization
Food Security Vulnerability [20]	Food sector	Wheat import dependency ratio in 2020 (in %) = (import – exports)/(domestic production + imports – exports) × 100 Cereal import dependency ratio in 2020 (in %) = (import – exports)/(domestic production + imports – exports) × 100 Trade balance of agricultural products in 2020 (in million US$) = (total export value of agricultural products – total import value of agricultural products) Prevalence of moderate or severe food insecurity in the total population 2018–2020 average (in %) Prevalence of under-nourishment 2018–2020 average (in %) Political stability and absence of violence terrorism (index)	Min. value = −47.1% (Kazakhstan); max. value = 100% (Congo) Min. value = −66.4% (Kazakhstan); max. value = 93.1% (Libya) Min. value = −10,789 million US$ (Bangladesh); max. value = 3049 million US$ (Turkey) Min. value = 2.3% (Kazakhstan); max. value = 88.3% (Congo) Min. value = <2.5% (Turkey); max. value = 41.7% (D.R. Congo) Min. value = −2.57 (Libya); max. value = 0.29 (Belarus)	No weighting, combination and standardization
Import Vulnerability Index [21]	Defense-related industry	Index 1 = Imports/(production − exports) Index 2 = Index 1 x (Defense procurement/domestic production) Index 3 = Index 2 ⨯ weighted country risk score Index 4 = Index 3 x growth in import share	Index 1: Minimum value in 1987 for diodes = 4.1%; max. value in 1987 for computers = 47.9% Index 2: Minimum value in 1982 & 1987 for diodes = 0.6%; max value in 1987 for capacitors = 12.3% Index 3: Minimum value in 1982 for diodes = 2.3%; max. value in 1987 for capacitors = 18.5% Index 4: Minimum value in 1987 for diodes = 2.9%; max. value in 1987 for capacitors = 17.0%	Subjective weighting and no standardization

Gupta [16] presented a vulnerability assessment related to the energy sector by combining three supply risk indicators and four market risk indicators into a composite index of energy vulnerability using PCA to assess the relative oil vulnerability of 26 net oil-importing countries for 2004. A higher index indicates higher vulnerability. Net oil import dependence is defined as the ratio of net oil imports (defined as the sum of the net crude oil imports and net refining product imports) to the oil supply (defined as the sum of crude oil domestic production and net oil imports). To measure oil vulnerability, the author used indicators such as the ratio of value of oil imports to gross domestic product (GDP), oil consumption per unit of GDP, GDP per capita and oil share in total energy supply, ratio of domestic reserves to oil consumption, exposure to geopolitical oil market concentration risks as measured by net oil import dependence, diversification of supply sources, political risk in oil-supplying countries, and market liquidity. The oil vulnerability index captured four values, ranging from the least vulnerable to the most vulnerable. Other studies in the energy sector followed a similar approach [22,23].

One conceptual refinement of an economic vulnerability index^2^ that is applicable to a whole economy consists of three subindices: import vulnerability, export vulnerability, and foreign direct investment vulnerability [17]. Each of the three subindices considers further variables depicting vulnerability. The subindices of import and export vulnerability consider the same variables. However, the variables country origin and import concentration are used for the subindex export vulnerability, whereas the variables country of destination and export concentration are used for the import vulnerability subindex. The foreign direct investments vulnerability subindex comprises the variables lack of foreign direct investment potential and country of origin. For some variables, the authors of the index used equal weights to combine the variables into a subindex (i.e., equal weights were assigned for the variables merchandise/service, import/export vulnerability and foreign direct investment vulnerability), whereas for others, different weights were assigned. In particular, different weights were used to aggregate the merchandise export vulnerability subindex and the services export vulnerability subindex into a single export vulnerability subindex. The same was true for the import vulnerability subindex, for which the weights were derived from the respective ratios of merchandise and services in total exports or imports, depending on the aggregated subindex. Similarly, different weights were used to aggregate the three subindices into the composite economic vulnerability index. The composite index of economic vulnerability for a given country was standardized by taking a value between 0 (no vulnerability) and 1 (high vulnerability). The empirical findings for 180 countries confirmed that small-state and least-developed countries, in particular, exhibited higher degrees of economic vulnerability than high-income countries.

The Bonilla index assesses the vulnerability of a country's food sector. In its basic form, the index is calculated as the ratio between the value of food imports (quantity of food imports times world price in foreign currency) and the value of total exports (quantity of exports times export price in foreign currency) [18]. Given that the Bonilla index is a single measure given in percentage values, no weighting or standardization is necessary. The food sector is less vulnerable to trade when the Bonilla index decreases and more vulnerable when the Bonilla index increases (i.e., low or moderate Bonilla index = 1–10%; high Bonilla index = 11–100%; Bonilla index >100% possible). In a methodological refinement of the Bonilla index, the authors introduced the nominal exchange rate and the nominal rate of the assistance index^3^ for importable food products [24]. The refined Bonilla index was calculated for 39 developing countries from 1995 to 2010 and for the 2008 food crisis at the HS 4-digit level.

The trade vulnerability of China in the international grain trade has also been assessed [19]. In the study, the vulnerability index was calculated by dividing a measure of import dependence by a measure of export dependence. Import dependence was measured as the share of the amount of China's grain imports from a given exporter (numerator) and the sum of China's grain imports from all exporters (denominator). In the same logic, export dependence was measured as the share of the amount of China's grain exports to a given importer (numerator) and the sum of China's grain exports to all importers (denominator). The values of import and export dependence ranged from 0 to 100%. Therefore, weighting and standardization of individual indicators (i.e., import and export dependence) was not necessary. With regard to import and export dependence, the results indicated that China's trade partners were of four country groups: highest dependence (≥15%), higher dependence (5%–15%), moderate dependence (1%–5%), and lower dependence (≤1%). The vulnerability index was used to analyze and characterize the spatial differences in China's grain trade vulnerability. The authors applied the natural fracture method to divide the vulnerability index into the following three county-level categories: low vulnerability (-∞, 0.7), medium vulnerability (0.7, 1.6), and high vulnerability (1.6, +∞).

A recent study assessed the food security vulnerability of 20 countries that were dependent on imports from Russia and Ukraine [20]. A country's vulnerability was measured based on three indicators capturing import dependence (i.e., wheat and cereal import dependency ratio and trade balance of agricultural products) and three indicators depicting coping capacities (i.e., prevalence of moderate and severe food insecurity in the total population, prevalence of undernourishment, and political stability). Countries that were not dependent on wheat and cereal imports from Ukraine and Russia exhibited negative import dependency values, whereas countries that were dependent showed positive values. The trade balance of a country is positive if the country imports more than it exports (and vice versa). The coping capacity indicators “prevalence of moderate and severe food insecurity in total population” and “prevalence of undernourishment, and political stability” theoretically ranged from 0 to 100%. The political stability indicator ranged from −4 to 2, with higher values reflecting better situations. The individual indicators were not combined into a standardized and composite vulnerability index.

A product-specific import vulnerability assessment of defense-related systems and subsystems (computers, storage devices, capacitors, diodes, transistors, semiconductors, and printed circuit boards) for the United States has been presented [21]. For this purpose, the authors derived four different import vulnerability indices, with higher index values indicating higher vulnerability. The most basic one was “IVI 1” (the import ratio), which was calculated by dividing the value of imports (in US$) by the value of domestic production (in US$) minus the value of exports (in US$). The other indices captured other variables, such as the import ratio (in %), the defense-related procurements (in US$), and the weighted country risk score of the supplying countries. Each variable was assigned a particular weight, summing up to one. For the empirical exercise, the authors assigned equal weights to each of the considered variables. The four different import vulnerability indices were computed for the years 1982 and 1987. The results indicated that the import vulnerability for most of the considered products increased from 1982 to 1987.

## 3 Conceptual framework, data bases, and methods

### 3.1 Conceptual framework for a food import vulnerability index and underlying data bases

The literature review on vulnerability assessments conducted in Section 2 revealed that a holistic, standardized, and combined vulnerability index in the realm of food trade is missing. Nevertheless, the literature review exhibited a variety of relevant factors for measuring food import vulnerability. In particular, factors (subindices) capturing the political risks of the supplying countries, the diversification of supply sources, import dependency, and the (volatility of) import prices can be incorporated in the design of a multifactorial and standardized IVI in the food sector. However, some relevant factors influencing agricultural production and the resulting supply capacity are not considered in the reviewed literature (i.e., climate risks and technological development). Therefore, our study contributes to the academic literature by determining food import vulnerability using a combination of existing individual exporter- and product-specific factors and relevant unexplored individual factors.

To capture the factors determining food import vulnerability, we initially examined relevant databases by focusing on the reliability and free availability of the data source. Subsequently, based on the available data, we conceptualized the IVI by linking its individual factors with import flows. The applicability of the proposed IVI to other countries is the main advantage of our approach.

**Fig. 1:**
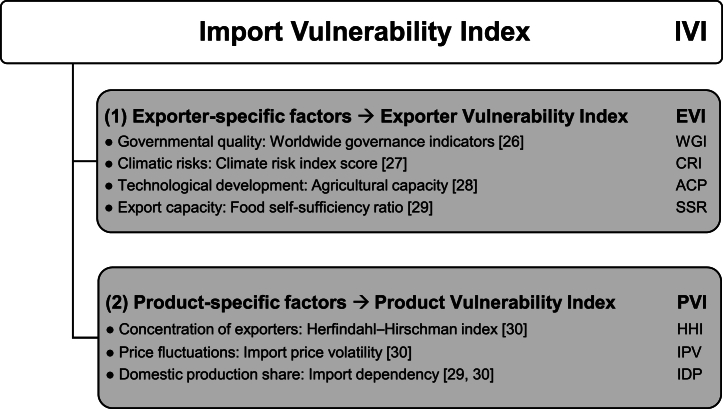
The multifactorial import vulnerability index consisting of four exporter/country of origin-specific and three product-specific factors.

Fig. 1 presents the conceptual framework of the IVI, which consists of factors already considered in the reviewed literature and those we introduced in this study. Our multifactorial IVI consists of (1) four exporter/country of origin-specific factors, resulting in the subindex EVI, and (2) three product-specific factors, resulting in the subindex PVI. The single factors are described in the following sections. To aggregate the factors into the subindices and the overall IVI, they have to be normalized and weighted (see subsection 3.2).

#### 3.1.1 Exporter-specific factors

The reliability of trading partners is indispensable for countries with a low food SSR. Exporter-specific factors depict conditions in a country of origin that influence agricultural production and trade infrastructure, which, in turn, affect the vulnerability of trade. Exporter-specific factors include governmental quality, exposure to climatic risks, export capacity, and the technological development of the agricultural sector.

Governmental quality is an important factor in a nation's economic performance [25]. Empirical evidence indicates that nations with a high quality of political institutions exhibit better economic performance than nations with a low institutional quality [26]. Accordingly, we assumed that food exporters with a high governmental quality are less vulnerable to economic shocks and are therefore more reliable. Climate change and associated extreme weather events (e.g., heat, drought, and storms) have increasingly negative impacts on agricultural production [27]. Here, the institutional quality of a nation plays a major role in mediating the transformation of coping capacity into adaptive capacity [28]. Due to a lack of adaptive capacity, agricultural production in developing countries is especially vulnerable to climate change [29]. Consequently, we assume that exporters that are less vulnerable to climatic risks are more reliable trade partners than countries that are more vulnerable. Technological progress has consistently increased yields and net output in the agricultural sector [30]. Therefore, we assume that countries characterized by a high level of technological progress in the agricultural sector exhibit higher export capacities than countries with a low level of technological progress. In this context, food-exporting countries usually have a production surplus that exceeds the domestic population's demand [15]. In the event of severe shortages, production in low-surplus countries could fall below home consumption more quickly than in high-surplus countries. Therefore, we assume that imports from countries with low surpluses are more vulnerable than imports from countries with high surpluses.

We selected the Worldwide Governance Indicators (WGI) developed and maintained by the World Bank [31] to measure the governmental quality of exporting countries. The WGI comprise the following six dimensions of governance: (1) voice and accountability, (2) regulatory quality, (3) political stability and absence of violence/terrorism, (4) rule of law, (5) government effectiveness, and (6) control of corruption. A detailed description of the six indicators was previously published [32]. Data are available for 200 countries and territories over the 1996–2020 period. WGI values range between −2.5 (weak governance performance) and +2.5 (strong governance performance). For the empirical application, we computed the average WGI value across all six dimensions.

To capture the exposure to climatic risks of a country of origin, we used the climate risk index (CRI) score, which has been published annually since 2006 [33]. The overall CRI score of a country is derived from its average ranking of four indicators, according to the following weighting: number of deaths because of climatic events, one-sixth; number of deaths per 100,000 inhabitants because of climatic events, one-third; sum of losses in US$ in purchasing power parity because of climatic events, one-sixth; and losses per unit of GDP because of climatic events, one-third. The lower the CRI score, the higher a country's exposure to climatic risks (and vice versa).

To assess a country's stage of technological development in the agricultural sector, we used the agricultural capacity indicator (ACP) [34]. This indicator is built by a combination of the following four indicators measuring agricultural technology: (1) capacity to equip agricultural areas with irrigation, (2) nitrogen plus phosphate total fertilizer use on arable and permanent crop areas, (3) pesticide use, and (4) tractor use. Data are available for 181 countries from 1995 to 2019. The agricultural capacity values range from 0 (“very high agricultural capacity”) to 1 (“very low agricultural capacity”).

To assess a country's export capacity, we used the indicator food self-sufficiency ratio (SSR) [35]. This database is available for 98 product categories and 170 countries [36]. We calculated the SSR according to Equation (1) by multiplying all product quantities in the numerator and denominator with their associated calorie contents (kcal)^4^:

(1) $$SSR=\frac{\sum_{\mathrm{pr}=1}^{n}\left({Production}_{\mathrm{pr}}\;⨯\;{kcal}_{\mathrm{pr}}\right)}{\sum_{\mathrm{pr}=1}^{n}\left({Domestic\;supply\;quantity}_{\mathrm{pr}}\;⨯\;{kcal}_{\mathrm{pr}}\right)}⨯\;100$$

#### 3.1.2 Product-specific factors

Product-specific factors directly refer to the import vulnerability of individual products. We assessed product-specific risks based on the concentration of exporters, import price volatility, and import dependence.

A high concentration of exporters for a given product category destabilizes trade. The food supply of an importing country is in danger when an exporter with a high share of total imports ceases export [37]. Therefore, we assume that product categories with a high concentration of exporters are more vulnerable than product categories with diverse exporting countries. Import price volatility for a specific product (i.e., price fluctuations or the risk of large, unexpected price changes) can intensify social vulnerability in terms of the food security, human development, and political stability [38]. A high import dependency for a specific product increases a country's vulnerability to external shocks, such as (import) price volatility or export bans [39].

The calculation of these factors was mainly based on trade statistics (volume and value of imports and unit values of imports), as provided by the Swiss Customs Administration [40], and partially on domestic production/consumption data provided by Ref. [36]. The product-specific factors were calculated based on the HS 2-digit level (product group level) for the years 2014–2019.

We used the Herfindahl−Hirschman index (HHI) to measure the concentration of exporting (supplying) countries for a given product category [41]. The HHI was calculated as the sum of squared market shares according to Equation (2):

(2) $$HHI=\sum_{e=1}^{\;n\;}{\left(d_{e}\right)}^{2}$$

whereby n represents the number of exporting countries for a given product group and $d_{e}$ the share of exporter e in a given product group.

We used the standard deviation as a measure of import price volatility (IPV; [42]) according to Equation (3):

(3) $$IPV=\sqrt{\frac{1}{n\;-\;1}\;\sum_{i=1}^{\;n}{\left(p_{m}-\;\bar{p}\right)}^{2}}$$

We computed the annual standard deviation of import prices based on the HS 2-digit level by using monthly trade data. Therefore, *n* represents the number of observations (*n* = 12 months), $p_{m}$ depicts the import price (unit value in CHF per kg) in month *m*, and $\bar{p}$ is the average import price per year. A high standard deviation can be considered an indication of a high IPV.

Import dependency (IDP) and import vulnerability can be measured in several ways (see Table 1). For an easy and understandable value, we assessed IDP as the share of food imports in relation to domestic supply. Given that imports and exports are likely to be simultaneously affected in the case of trade disruptions, net imports were considered according to Equation (4):

(4) $$IDP=\frac{\left(Imports\;⨯\;kcal\;-\;Exports\;⨯\;kcal\right)}{\left(Domestic\;supply\;quantity\;⨯\;kcal\right)}$$

The quantities of net imports and domestic supply were multiplied by their associated kilocalories (kcal). Data on product level [36] were assigned to the most appropriate 2-digit level HS chapter. Higher IDP values corresponded to a high degree of import dependency (and vice versa). Values of the import dependency for a specific product can become negative when exports are larger than imports (negative factor values are converted into values within the range from 0 to 1 via the normalization formulas; see Equations (5), (6)) in subsection 3.2).

### 3.2 Method: construction of the composite import vulnerability index using principal component analysis

The literature review revealed multiple methods of combining individual factors into an overall vulnerability index: (i) further weighting variables [17], (ii) subjective weights [21], or (iii) no weights are used [[18], [19], [20]]. To overcome these methodological shortcomings, we used PCA to determine the weights for combining these individual factors into a standardized IVI, as previously suggested [16,22,23]. In contrast to ordinary PCA, the minimum covariance determinant (MCD)-based robust variant of PCA can provide more reliable and stable results when outliers are frequent in the data [43]. The IVI and its subindices (EVI and PVI) can systematically capture the interrelated factors that make food imports vulnerable, while a higher value of the IVI and its subindices will indicate a higher vulnerability. In contrast to subjective or equal weighting, PCA is a multivariate method that can derive the weights of individual exporter- and product-specific factors.

Before conducting the PCA, an imputation of missing values was necessary for both the CRI score (approximately 3% missing values) and agricultural capacity (approximately 2% missing values). For this, we used the k-nearest neighbor classification based on a variation of the Gower distance [44]. Following previous studies [16,22,23], all individual factors were normalized in such a way that the values of all factors ranged between 0 “low (import) vulnerability” to 1 “high (import) vulnerability.” Normalizing ensures that all factors are scaled the same. Some factors were already positively related to import vulnerability (agricultural capacity, HHI, import dependency, and IPV), whereas others were negatively related to import vulnerability (WGI, CRI score, and food SSR). For already positively related factors, we used the formula in Equation (5) for value normalization [45]:

(5) $$x_{pt}=\frac{\;X_{pt}-\;\mathrm{Min}\left(X_{pt}\right)}{\mathrm{Max}\left(X_{pt}\right)-\mathrm{Min}\left(X_{pt}\right)}$$

where the subscript p represents factors already positively related to import vulnerability (agricultural capacity, HHI, import dependency, and IPV), and t is the time (years 2014–2019).

For negatively related factors, we performed value normalization using Equation (6):

(6) $$x_{zt}=\frac{\mathrm{Max}\left(X_{zt}\right)-\;X_{zt}}{\mathrm{Max}\left(X_{zt}\right)-\mathrm{Min}\left(X_{zt}\right)}$$

where the subscript z represents factors negatively related to import vulnerability (WGI, CRI score, and food SSR), and t is the time (years 2014–2019).

Table 2 presents the summary statistics of the normalized exporter- and product-specific factors.

**Table 2:** Summary statistics of normalized exporter- and product-specific factors.

Factor		Mean	Std. Dev.	Skewness	Kurtosis
Worldwide governance indicators	WGI	0.49	0.22	−0.36	2.46
Climate risk index score	CRI	0.39	0.27	0.23	1.94
Agricultural capacity	ACP	0.80	0.23	−1.5	4.65
Food self-sufficiency ratio	SSR	0.69	0.17	−0.93	4.18
Herfindahl–Hirschman index	HHI	0.35	0.24	0.98	3.47
Import price volatility	IPV	0.18	0.17	1.79	7.32
Import dependency	IDP	0.37	0.24	0.05	2.31

We applied a robust PCA based on a robust covariance estimation. PCA is a multivariate method that can transform a set of correlated variables into a set of uncorrelated variables called components. The uncorrelated components represent the linear combinations of the original variables. The first component is a linear function that has the maximum possible variance. The second component is a linear function with the maximum possible variance, being uncorrelated with the first component; the third component is a linear function that maximizes variance, being uncorrelated with the first and second principal components [46]. The main goal of conducting PCA is to statistically derive the weights of individual factors [47]. This is based on the consideration that individual factors contribute differently to the overall vulnerability index.

For each year, we separately applied a robust PCA for exporter-specific factors, resulting in an EVI, and for each product group (rsp. HS chapter), we applied a robust PCA for product-specific factors, resulting in a PVI. Both the EVI and the PVI were finally combined into a standardized IVI. In the following, we describe the construction of the EVI, the PVI, and the overall combined IVI based on PCA [45].

In the first step, we computed the MCD estimator to obtain a robust location and scatter estimation [48]. The loading matrix was obtained by a standardized eigenvalue decomposition of the MCD covariance matrix, as shown in Equation (7):

(7) $${Cov}_{MCD}=\Lambda_{MCD}C_{MCD}\Lambda_{MCD}^{\prime }$$

with the matrix of eigenvectors $C_{MCD}$, which is the loading matrix, and the diagonal matrix $\Lambda_{MCD}$, which contains the eigenvalues in its diagonal. These eigenvalues were the (robust) variances of the principal components [49]. By using robust variances, the most important principal components summarize the information described by the joint distribution of the majority of the data. Non-robust PCA can result in principal component directions that are attracted by outliers. This may lead to a large (non-robust) influence. Therefore, the variance of non-robust principal components should not be used to identify outliers, as identifying outliers is not the purpose of PCA [49].

The eigenvalues λ, the variability (in %), and the cumulative variance explained (in %) are shown in Table 3 for exporter-specific factors and in Table 4 for product-specific factors.

**Table 3:** Eigenvalues, variability (in %), and cumulative variance explained (in %) for exporter-specific factors.

	$\lambda_{1}$	$\lambda_{2}$	$\lambda_{3}$	$\lambda_{4}$
Eigenvalue	1.4	1.1	1.0	0.5
Variability (%)	35.0	27.2	24.9	12.9
Cumulative Variance explained (%)	35.0	62.2	87.1	100.0

Note: In contrast to the method described above, these results are based on a PCA including observations of all years.

**Table 4:** Eigenvalues, variability (in %), and cumulative variance explained (in %) for product-specific factors.

	$\lambda_{1}$	$\lambda_{2}$	$\lambda_{3}$
Eigenvalue	1.5	1.0	0.5
Variability (%)	51	33.9	15.1
Cumulative Variance explained (%)	51	84.9	100.0

Note: In contrast to the method described above, these results are based on a PCA including observations of all years and all product groups.

Based on the absolute value of factor loadings and variance explained in the form of eigenvalue, the coefficient w for each exporter- and product-specific factor was determined through Equation (8), that is, the weights of the vulnerability indicators (Appendix, Tables 11 and 12):

(8) $$w_{ij}=\frac{\left|c_{i}\right|\lambda_{i}}{\sum_{i=1}^{n}\sum_{j=1}^{m}\left|c_{i}\right|\lambda_{i}}$$

where n represents the number of indicators, and m is the number of principal components selected from the variance explained, with only principal components with an eigenvalue greater than or equal to 1 selected. $C_{i}$ depicts the principal component loadings of the i ^th^ indicator, and $\lambda_{j}$ is the variance explanation of the j th principal component. The rotated component matrix for exporter-specific factors is presented in Table 5, and the rotated component matrix for product-specific factors is presented in Table 6.

**Table 5:** Rotated component matrix for exporter-specific factors.

Factor	$C_{1}$	$C_{2}$	$C_{3}$
WGI	0.708		0.275
CRI		0.817	0.461
ACP	0.705	−0.148	−0.258
SSR		−0.550	0.803

Note: In contrast to the method described above, these results are based on a PCA including observations of all years.

**Table 6:** Rotated component matrix for product-specific factors.

Factor	$C_{1}$	$C_{2}$
HHI	0.710	0.100
IPV		0.981
IDP	−0.703	0.164

Note: In contrast to the method described above, these results are based on a PCA including observations of all years and all product groups.

In the next step, the coefficients w estimated by Equation (8) were used to calculate the EVI (Equation (9)) and the PVI (Equation (10)):

(9) $${EVI}_{et}=w_{1}{WGI}_{et}+w_{2}C{RI}_{et}+w_{3}{ACP}_{et}+w_{4}{SSR}_{et}$$

(10) $${PVI}_{gt}=w_{1}{HHI}_{gt}+w_{2}{IPV}_{gt}+w_{3}{IDP}_{gt}$$

where subscript e denotes an exporting country, g the product group, and t the time (years 2014–2019). Each coefficient w represents the weight of its corresponding normalized factor in the EVI and PVI.

In the final step, the EVI and PVI were combined into the overall IVI. The aggregated value consists of the mean value of the PCA-weighted EVI across all countries from which at least one product group was imported and of the mean value of the PCA-weighted PVI across all HS chapters (Equation (11)). Weighting with the import shares was not implemented because the import shares would have dominated the aggregated value. Due to the absence of weighting by import shares, the effects of changing vulnerability factors can be distinguished from the effects of changing import shares. For the same reason, no weighting or equal weighting of the EVI and PVI was chosen.

(11) $${IVI}_{t}=\frac{1}{n}\;\sum_{e=1}^{n}{EVI}_{et}+\frac{1}{n}\;\sum_{g=1}^{n}{PVI}_{gt}$$

In addition, a product-specific IVI was calculated. For this purpose, the mean EVI value across all countries from which the corresponding product group was imported was added to the existing PVI value of the product group (Equation (12)).

(12) $${IVI}_{gt}=\frac{1}{n}\;\sum_{e=1}^{n}{EVI}_{et}+{PVI}_{gt}$$

## 4 Empirical results

In subsection 4.1, we present the results of the EVI, and in subsection 4.2, we provide the results for the PVI. The results for the overall (multifactorial) IVI are presented in subsection 4.3.

### 4.1 Exporter vulnerability index

**Fig. 2:**
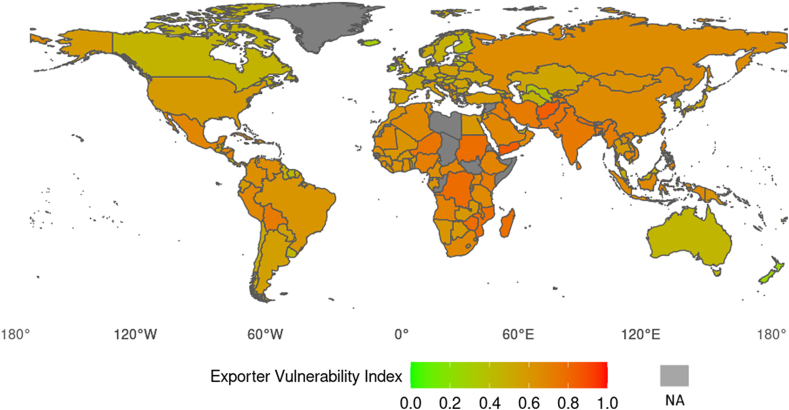
Average value for the exporter vulnerability index from 2014 to 2019 for each exporting country.

For each exporting country, the average value for the EVI from 2014 to 2019 is visualized on the world map depicted in Fig. 2. All potential countries exporting to Switzerland were considered, even those with a very low share of the total Swiss food imports. (Note that nearly every country depicted on the world map exported to Switzerland from 2014 to 2019.) The EVI ranged between 0 (“very low exporter vulnerability index”) (light green color) and 1 (“very high exporter vulnerability index”) (red color). Countries without exports to Switzerland or countries with no available EVI factor values in the data sources are colored gray.

To provide a clearer picture of the relevance of exporting countries, their EVI values, and their associated share of total food imports, we computed country clusters based on quintiles of the average EVI values. The results of the cluster analysis, which considered EVI values in combination with volumes of trade, are presented in Table 7. (The results of the cluster analysis considering EVI values in combination with values of trade are presented in Table 13 in the appendix.) The lowest EVI value obtained was 0.26 (New Zealand; share total imports: 0.2%), and the highest was 0.85 (Yemen; share total imports: 0.001%).

**Table 7:** Clusters computed based on quintiles of the exporter vulnerability index from 2014 to 2019.

Country group	No. countries	Exporter Vulnerability Index from 2014 to 2019			Share volume total food imports from 2014 to 2019 (in %)			
		Min.	Max.	Average	Min.	Max.	Average	Sum
1 “Very low”	33	0.26	0.49	0.43	2.2^–7^	3.7	0.3	9.1
2 “Low”	32	0.49	0.57	0.53	1.0^–6^	21.7	2.4	76.3
3 “Moderate”	33	0.57	0.63	0.60	2.4^–6^	2.9	2.3	7.5
4 “High”	32	0.63	0.68	0.66	2.8^–6^	1.1	1.4	4.6
5 “Very high”	33	0.69	0.85	0.74	1.4^–6^	0.8	0.1	2.5

The EVI of Group 1 countries ranged between 0.26 and 0.49. Accordingly, this country group indicated very low vulnerability. The top three exporters in Group 1, as measured by the volume of imports into Switzerland, were the Netherlands (EVI: 0.37; share of total imports: 3.7%), Canada (EVI: 0.46; share of total imports: 1.3%), and Hungary (EVI: 0.45; share of total imports: 1.0%). From 2014 to 2019, the countries in Group 1 accounted for 9.1% of total food imports into Switzerland. By contrast, the EVI of Group 5 countries ranged between 0.69 and 0.85. This group exhibited very high vulnerability. The top three exporters in Group 5 were India (EVI: 0.74, share total imports: 0.8%), Vietnam (EVI: 0.69, share total imports: 0.5%), and Pakistan (EVI: 0.76, share total imports: 0.2%). From 2014 to 2019, the countries in Group 5 accounted for only 2.5% of the total Swiss food imports.

Approximately 76% of the total Swiss food imports originated from countries that exhibited low vulnerability. The EVI of Group 2, which included 32 countries, ranged between 0.49 and 0.57. The top three exporters in Group 2 were France (EVI: 0.51, share total imports: 21.7%), Germany (EVI: 0.52, share total imports: 21.3%), and Italy (EVI: 0.56, share total imports: 15.2%).

Table 8 shows the top 10 exporting countries (i.e., countries exporting food to Switzerland) from 2014 to 2019 and their country group membership. Regarding the volume of trade, the top 10 exporting countries accounted for approximately 80% of total food imports from 2014 to 2019. The results based on the value of trade (in CHF) for the top 10 exporting countries can be found in Table 14 in the appendix.

**Table 8:** Top 10 exporting countries from 2014 to 2019 (based on volume of trade).

Exporting country	Country group	Exporter Vulnerability Index	Share volume total food imports (in %)
France	2	0.51	21.7%
Germany	2	0.52	21.3%
Italy	2	0.56	15.2%
Spain	2	0.55	7.0%
Austria	2	0.52	4.3%
Netherlands	1	0.37	3.7%
Brazil	3	0.61	2.9%
Canada	1	0.46	1.3%
Belgium	2	0.50	1.2%
Colombia	4	0.64	1.1%

Two exporting countries showed a very low vulnerability (Group 1). From 2014 to 2019, these countries accounted for 5.0% of total food imports. Six exporting countries belonged to Group 2 (“low vulnerability”), accounting for 70.7% of total food imports. Only two countries, Brazil and Colombia, exhibited moderate to high vulnerability, accounting for 4.0% of the total food imports.

### 4.2 Product vulnerability index

**Fig. 3:**
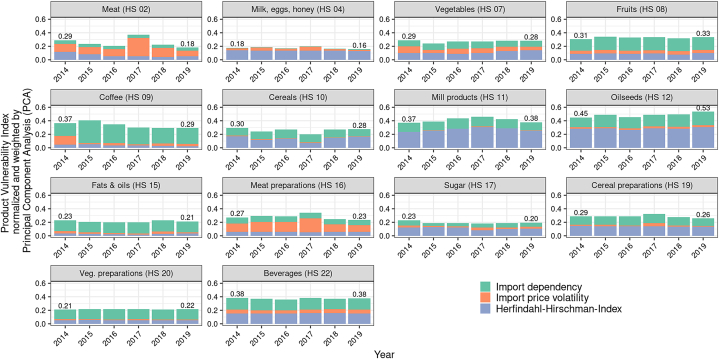
Results for the product vulnerability index (PVI) per product group and year.

For most of the considered product groups, the PVI value remained relatively stable from 2014 to 2019 (Fig. 3). Only for the product group HS 12 “Oilseeds” did we observe a moderate increase in the PVI from 2014 to 2019, resulting mainly from an import dependency, which fluctuated but tended to increase. For the product group HS 11 “Mill products,” the PVI value increased until 2017 because of a decreasing number of countries from which this product group was imported but dropped afterwards. In 2017, we observed a high import price vulnerability for meat (HS 02 “Meat” and HS 16 “Meat preparations”), likely because of price fluctuations in import quotas auctioned in Switzerland. A slightly decreasing trend has been observed for HS 09 “Coffee” since 2015.

Table 9 depicts the average PVI values from 2014 to 2019, as listed by product group and subdivided by individual PVI factors.

**Table 9:** Average PVI values per product group and average values of the individual PVI factors from 2014 to 2019.

HS chapter	PVI value	HHI value	IPV value	IDP value	Share HHI of PVI (in %)	Share IPV of PVI (in %)	Share IDP of PVI (in %)
**02** Meat	0.25	0.07	0.14	0.05	26.2	54.8	19.0
**04** Milk, eggs, and honey	0.18	0.13	0.04	0.01	76.5	20.0	3.4
**07** Vegetables	0.27	0.11	0.07	0.09	40.5	25.0	34.6
**08** Fruits	0.33	0.09	0.05	0.19	27.5	15.2	57.4
**09** Coffee	0.33	0.04	0.05	0.25	11.2	13.5	75.3
**10** Cereals	0.26	0.13	0.01	0.12	51.5	3.8	44.7
**11** Mill products	0.41	0.27	0.00	0.14	66.0	0.9	33.2
**12** Oilseeds	0.49	0.29	0.02	0.18	58.7	5.1	36.2
**15** Fats & oils	0.21	0.03	0.02	0.16	13.1	11.5	75.4
**16** Meat preparations	0.28	0.06	0.14	0.08	20.7	49.8	29.5
**17** Sugar	0.20	0.11	0.03	0.06	56.6	13.3	30.1
**19** Cereal preparations	0.29	0.14	0.02	0.13	48.6	7.6	43.8
**20** Vegetable preparations	0.22	0.05	0.01	0.15	24.6	5.9	69.5
**22** Beverages	0.37	0.16	0.05	0.16	41.5	14.5	43.9

HS: Harmonized System of tariff nomenclature, PVI: product vulnerability index, consisting of HHI (Herfindahl–Hirschman index), IPV (import price volatility), and the IDP (import dependency).

From 2014 to 2019, with an average PVI value of 0.18, the product group HS 04 “Milk, eggs, and honey” showed the lowest vulnerability. The factor IDP (average value = 0.01) accounted for, on average, 3.4% of the total PVI value of HS 04, followed by the factor IPV (average value 0.04) with, on average, 20%, and the factor HHI (average value 0.13) with, on average, 76.5%. Regarding volume of trade, HS 04 accounted for 3.0% of total food imports, and the value of trade accounted for 6.7% (see Table 15 in the appendix).

By contrast, with an average PVI value of 0.49, the product group HS 12 “Oilseeds” showed the highest vulnerability. The average PVI value of HS 12 was mainly determined by a market concentration that was above average. The corresponding factor HHI (average value = 0.29) accounted for, on average, 58.7% of the total PVI value, followed by the factor IDP (average value 0.18), with, on average, 36.2%, while the import price vulnerability factor IPV hardly seemed to play a role (average value = 0.02, with, on average, 5.1%) for HS 12.

The product group HS 11 “Mill products” exhibited a comparatively high PVI, with an average value of 0.41. This was mainly driven by the HHI factor. The HHI (average value = 0.27) accounted for, on average, 66% of the total PVI value, followed by the factor IDP (average value 0.14), with, on average, 33.2%, and the factor IPV (average value = 0.00), with, on average, 0.9%. Regarding volume of trade, HS 11 accounted for only 2.7% of the total food imports, and the value of trade was 1.0% (see Table 15 in the appendix).

Regarding the share of total imports, the most important product group HS 22 “Beverages” (share total trade volume: 23.3%; share total trade value: 21.5%) had a moderate average PVI value of 0.37. The average PVI value of HS 22 was again mainly determined by the factors of market concentration (HHI) and import dependency. The factor HHI (average value = 0.16) accounted for, on average, 41.5% of the total PVI value, and the factor IDP (average value 0.16) accounted for, on average, 43.9%, followed by the factor IPV (average value = 0.05), with an average dominance of 14.5%.

With an average HHI value of 0.29, HS 12 “Oilseeds” exhibited the largest market concentration, followed by HS 11 “Mill products” (average HHI value = 0.27) and HS 22 “Beverages” (average HHI value = 0.16), whereas the lowest (average HHI value = 0.03) was observed for HS 15 “Fats and oils.” Product group HS 16 “Meat preparations” showed the highest average IPV (0.14), whereas the import prices in product group HS 11 “Mill products” were stable (average IPV = 0.00). HS 09 “Coffee” and HS 08 “Edible fruits and nuts” showed the highest import dependency (average IDP HS 09 value = 0.25 and average IDP HS 08 value = 0.19), whereas HS 04 “Milk, eggs, and honey” had the lowest (average IDP value = 0.01).

### 4.3 Overall import vulnerability index (IVI)

**Fig. 4:**
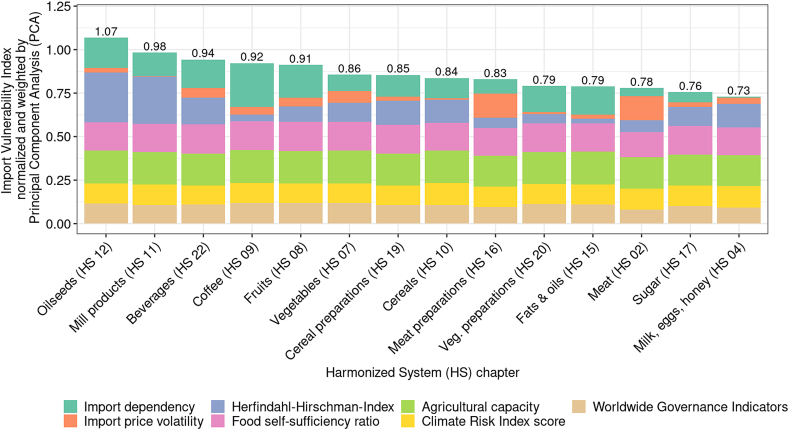
Average import vulnerability index values and average values of its individual factors per product group (2014–2019).

Fig. 4 shows the average IVI values for the period 2014–2019, including the values of its individual factors per product group. The individual factors consist of four factors related to the EVI (i.e., worldwide governance indicators, climate risk index, agricultural capacity, and self-sufficiency ratio) and three factors related to the PVI (Herfindahl-Hirschman index, import price volatility, and import dependency). Accordingly, the overall IVI at the product group level was calculated as the sum of the individual factors.

With an average IVI value of 1.07, product group HS 12 “Oilseeds” indicated the highest vulnerability, followed by product group HS 11 “Mill products” (average IVI value = 0.98) and product group HS 22 “Beverages” (average IVI value = 0.94). The HHI mainly determined the overall IVI for all six product groups, whereas the IPV was the main determinant of the overall IVI for HS 02 and HS 16. For HS 2, the IDP and HHI played a crucial role in determining the overall IVI. With an average IVI value of 0.73, the product group HS 04 “Milk, eggs, & honey” exhibited the lowest vulnerability, whereby individual factors of the EVI were more important in the composition of the overall IVI than individual factors of the PVI.

In the composition of the overall (product-group-specific) IVI, the individual factors of the EVI were, for most of the product groups, more relevant than the individual factors of the PVI. For all product groups, the contribution of the individual EVI factors (WGI, CRI score, food SSR, and agricultural capacity) to the overall IVI was spread out and relatively even. For two product groups HS 11 and HS 12, we observed that the market concentration (HHI) accounted for the largest share of the overall IVI. The two product groups “Coffee” (HS 09) and “Edible fruits and nuts” (HS 08) were most affected by import dependency, whereas the vulnerability of the other product groups was determined by production difficulties in exporting countries, whether because of a lack of supply capacity or reserves in technological development in the respective agricultural sector.

**Fig. 5:**
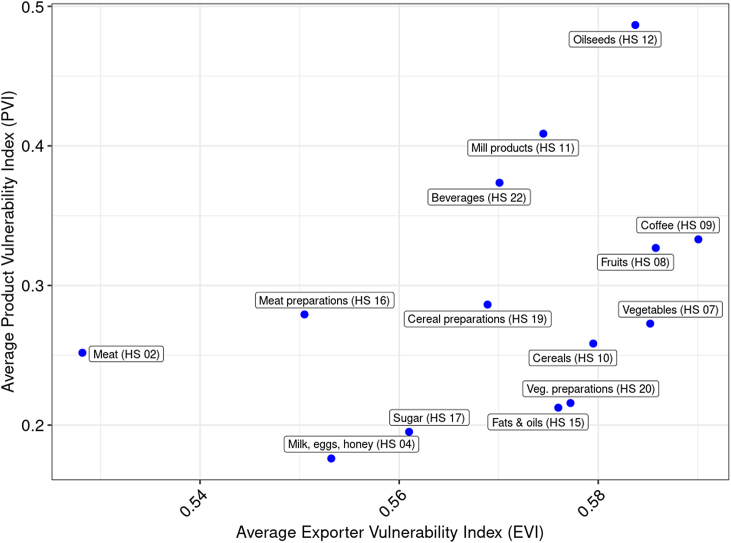
Relationship between product-group-specific product vulnerability index values and product-group-specific exporter vulnerability index values from 2014 to 2019.

In general, we found no linear relationship between product-specific average PVI values and product-specific average EVI values (Fig. 5; correlation coefficient *r* = 0.398; *p* = 0.158).

**Fig. 6:**
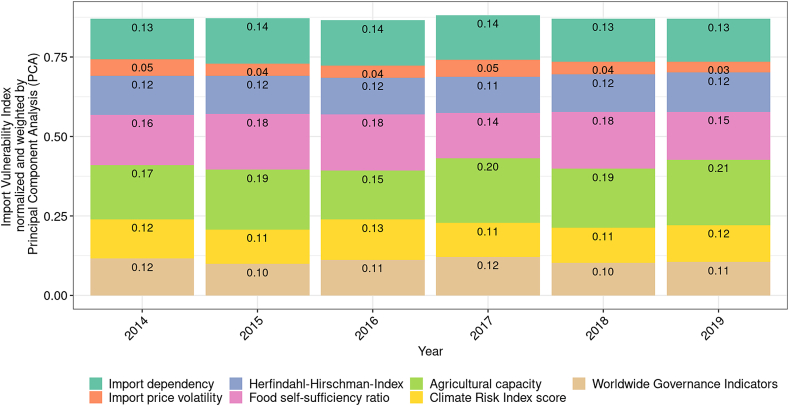
Development of the import vulnerability index (IVI) and its individual factors overall product groups from 2014 to 2019.

The single-score IVI exhibited neither large fluctuations nor a decreasing or increasing trend from 2014 to 2019 (Fig. 6). The minimum IVI value was observed for 2016 (IVI value = 0.86), and the maximum IVI value was observed for 2017 (IVI value = 0.88). Even though climate change increasingly negatively affects global agricultural production (World Bank, 2021), surprisingly, the contribution of the CRI to the IVI remained almost constant (CRI value = 0.12) to 2019 (CRI value = 0.13). In general, exporter-specific factors played a superordinate role compared with product-specific factors. The share of the product-specific factors of the IVI was between 33.6% and 34.9% from 2014 to 2019 (average share of product-specific factors of the IVI = 34.3%). In this context, with an average share of 21.6%, the IVI was mainly driven by the ACP, followed by the SSR (average share of the IVI = 19.1%) and IDP (average share of the IVI = 14.2%).

## 5 Conclusion and policy implications

The COVID-19 pandemic and the war in Ukraine have highlighted the vulnerability of global food supply chains. Against this background, it is critical for policymakers and food suppliers to gain empirical evidence regarding the vulnerability of food supply. By constructing a multifactorial and standardized IVI, we were able to assess the vulnerability of Swiss food imports on an aggregated product group level (HS 2-digit level) from 2014 to 2019. The IVI consists of four exporter-specific and three product-specific factors. The weights of the individual factors were estimated using a robust PCA.

Regarding exporting countries, the empirical results revealed that a large share of Swiss food imports originated from neighboring countries or countries with a low bilateral distance, which indicated low EVI values. For oilseeds and mill products, we observed a relatively high concentration of exporting countries. In this case, vulnerability could be reduced by enhancing the diversification of exporting countries. IDP was relatively high for coffee and fruits. Obviously, coffee and some exotic fruits cannot be produced in Switzerland, so import dependency cannot be reduced by increasing domestic production. Price volatility was an issue for meat and meat preparations, which were imported in auctioned tariff rate quotas. The auctions of tariff rate quotas might be considered a reason behind the price volatility. Moreover, we demonstrated that the IVI showed neither large fluctuations nor a decreasing or increasing trend from 2014 to 2019, indicating a resilient food supply.

Our proposed indicator was easily implementable but limited to 14 product groups and seven risk factors. However, our study has limitations. The validity and practicability of the indicator could be improved by further broadening the data basis. First, the product groups should be differentiated. Important stable foods, such as rice or palm oil, may have product properties and risk characteristics other than the whole product groups to which they belong. Second, more specific or other types of risks should be considered. For example, disruptions in transport routes and supply chains can have major effects on the timely delivery of the required food quantities, or the concentration of food production in a few large companies can threaten the food supply in the case of severe incidents or failure. Third, the import of production resources, such as seeds, fertilizers, and energy, should be included. Imported production resources increase the degree of foreign dependence on the domestic food supply. Fourth, indicators of domestic production also affect the vulnerability of the food system and, therefore, may also be considered within the framework of the whole indicator. Such domestic indicators include, for example, a decrease in agricultural land or the spatial concentrations of processing plants. Finally, these factors and indicators should be updated regularly to monitor and detect increasing vulnerabilities. Some figures may even be updated in real time. However, all of these enhancements require the availability of corresponding data. Although many public and private institutions provide such information, the establishment of a system for the easy collection and processing of incomplete and often difficult combinable data remains challenging.

The multifactorial and standardized IVI is suitable mainly for richer countries with low food self-sufficiency. It complements existing food security indicators that focus on monetary access to food. For the purpose of comparability and sensitivity, it should be applied to other countries. Hence, future research should focus on enhancing the indicator and its application to other countries.

## Data availability

The data that support the findings of this study are openly available in zenodo at http://doi.org/10.5281/zenodo.10682890.

## CRediT authorship contribution statement

**Christian Ritzel:** Writing – review & editing, Writing – original draft, Visualization, Validation, Supervision, Software, Project administration, Methodology, Investigation, Formal analysis, Data curation, Conceptualization. **Anke Möhring:** Writing – review & editing, Writing – original draft, Visualization, Validation, Software, Methodology, Investigation, Formal analysis, Data curation, Conceptualization. **Albert von Ow:** Writing – review & editing, Writing – original draft, Visualization, Validation, Software, Methodology, Investigation, Formal analysis, Data curation, Conceptualization.

## Declaration of competing interest

The authors declare that they have no known competing financial interests or personal relationships that could have appeared to influence the work reported in this paper.

## Figures and Tables

**Table 10:** HS chapters considered and not considered for the empirical

HS chapter	Description	Included in the analysis
01	Live animals	-*
02	Meat	02
03	Fish and crustaceans	-*
04	Milk, Eggs, Honey, etc.	04
05	Products of animal origin	-*
06	Live plants	-*
07	Edible vegetables	07
08	Edible fruits and nuts	08
09	Coffee	09
10	Cereals	10
11	Products of the milling industry	11
12	Oilseeds	12
13	Vegetable extracts	-*
14	Vegetable plaiting materials	-*
15	Fats and oils	15
16	Meat preparations	16
17	Sugar	17
18	Cocoa and cocoa preparations	-*
19	Preparations of cereals	19
20	Preparations of vegetables	20
21	Miscellaneous edible preparations	-*
22	Beverages	22
23	Animal feed (food industry residues)	-*
24	Tobacco	-*

* HS chapters are not considered in the calculation of the PVI because they contain hardly any food products (HS 01, 06, 13, 14, 23, 24), imports are very small (HS 05 and 21: 0.2% of Swiss food imports), or domestic production is constant at nearly 0% (HS 03 and 18; constant values are unfavorable for the PCA method).

**Table 11:** Weights of all EVI factors

**Year**	WGI	CRI	ACP	SSR	∑
2014	0.266	0.276	0.218	0.240	1
2015	0.231	0.263	0.242	0.264	1
2016	0.266	0.276	0.208	0.250	1
2017	0.282	0.240	0.262	0.216	1
2018	0.236	0.259	0.240	0.266	1
2019	0.244	0.266	0.264	0.225	1

**Table 12:** Weights of all PVI factors

HS chapter	HHI	IPV	IDP	∑
02	0.357	0.269	0.373	1
04	0.340	0.345	0.316	1
07	0.298	0.351	0.351	1
08	0.354	0.289	0.357	1
09	0.331	0.334	0.336	1
10	0.351	0.357	0.292	1
11	0.309	0.347	0.344	1
12	0.321	0.328	0.351	1
15	0.367	0.312	0.321	1
16	0.341	0.305	0.354	1
17	0.309	0.345	0.345	1
19	0.334	0.349	0.317	1
20	0.331	0.324	0.345	1
22	0.351	0.277	0.372	1

**Table 13:** Country clusters computed based on quintiles of the exporter vulnerability index from 2014 to 2019.

Country group	No. countries	Exporter Vulnerability Index from 2014 to 2019			Share value of total food imports from 2014 to 2019 (in %)			
		Min.	Max.	Average	Min.	Max.	Average	Sum
1 “very low”	33	0.26	0.49	0.43	1.1^–6^	5.5	0.4	12.6
2 “low”	32	0.49	0.57	0.53	7.3^–6^	16.0	2.1	66.2
3 “moderate”	33	0.57	0.63	0.60	1.1^–4^	3.0	0.3	10.6
4 “high”	32	0.63	0.68	0.66	5.7^–6^	1.3	0.2	6.0
5 “very high”	32	0.69	0.85	0.74	4.0^–6^	1.4	0.1	4.4

**Table 14:** Top 10 exporting countries from 2014 to 2019 (based on the value of the trade).

Exporting country	Country group	EVI	Share value total food imports (in %)
Italy	2	0.56	16.0
Germany	2	0.52	16.0
France	2	0.51	13.3
Spain	2	0.55	8.3
Netherlands	1	0.37	5.5
Austria	2	0.52	3.4
Brazil	3	0.62	3.0
United States	3	0.59	2.6
United Kingdom	2	0.51	1.9
Belgium	2	0.50	1.5

**Table 15:** Share of each product group of total imports from 2014 to 2019.

HS chapter	Description	Share volume 2014–2019 (in %)	Share value 2014–2019 (in %)
02	Meat	2.0	7.6
04	Dairy produce	3.0	6.7
07	Edible vegetables	6.3	7.1
08	Edible fruits and nuts	11.1	14.4
09	Coffee	3.7	9.4
10	Cereals	16.1	3.3
11	Products of the milling industry	2.7	1.0
12	Oil seeds	14.1	3.1
15	Fats and oils	3.4	4.1
16	Meat perpetrations	0.8	4.1
17	Sugars	4.3	2.8
19	Preparations of cereals	4.8	9.0
20	Preparations of vegetables	4.5	5.9
22	Beverages	23.3	21.5

## Footnotes

1 The other three dimensions are *economic and physical access to food*, *food utilization*, and *stability of the other three dimensions over time* [11].

2 Beside the GDP per capita and human asset index, the economic vulnerability index developed by the United Nations Committee for Development Policy (UNCDP) is used for the identification of the least-developed countries. The economic vulnerability index of the UNCDP consists of the following eight subindicators: (1) share of agriculture, forestry and fishing in GDP, (2) share of population in low elevated coastal zones, (3) remoteness and landlockedness, (4) share of population living in drylands, (5) merchandise export concentration, (6) instability of agricultural production, (7) instability of goods and services, and (8) victims of disasters. The economic vulnerability index ranges from 0 to 100, with a high score corresponding to a high level of vulnerability [50].

3 The nominal rate of assistance is defined as the percentage by which government policies have increased the revenues of domestic producers above the level they would be without the government's interventions (i.e., agricultural policy domestic support and border measures) [24].

4 The calorie contents that derive from the database are related to the share of the products used as food, whereas production, import and export quantities are only available for the total of all usages of the products. To aggregate these quantities of different products, the kcal unit is applied to the total of all usages. Furthermore, processed quantities are subtracted from the production quantity of a likely end product to prevent double counting. For this, a general scheme of processing coefficients and type of end products is used because country-specific factors are not available.
